# Medical Societies

**Published:** 1887-10

**Authors:** Z. T. Miller


### MEDICAL SOCIETIES: HOW TO MAKE THEM USEFUL.

Had I a spite at a man, I would persuade him to read a paper before an intelligent body during the month of August. I'd down him.

Upon this occasion these be my doctrines. Whether they be sound and well delivered or not is further on.

I want to speak of familiar things. I need not tell you that our County Society is, seemingly, starving to death. While the membership is good, the attendance is very bad as regards numbers. Now it behooves those of us who do attend with some degree of regularity to ask ourselves why it is that no more interest is taken in our meetings. When I first entered the ranks ALL the older and stauncher members came regularly. It's not so now, and why? Nature's inevitable has made vacant the places of Coté, Rousseau, Cowly, and, last, but by no means least, Caruthers. The night of death has gathered around them, but the morning of their memory is fair to those who loved them.

How of the living? Have they been crowded out? Have the vaporings of later accessions made it stale and unprofitable to spend one evening a month at a gathering of homœopathic physicians? Have the papers and discussions been so insipid that to stay at home brought better returns? Something is wrong. What is it? One thing is absolutely certain: Society attractions are not as great as they should be; they are not such as make it a pleasure, yea, a necessity, to attend; the feast spread must be inferior, else more would seek it. We are all hungry, because all of us flock to the annual dinner with royal precision.

Now it's the duty of every one of us to suggest a remedy for this lack of interest. Of course, no one will offer monthly *gastronomic* layouts as a solution to the problem, but I believe it opportune to submit that monthly layouts of mental pap prepared from experience resulting from the application of *Similia similibus curantur*, sweeted by the milk of human kindness and the honey of health restored, would attract each and every man who subscribes to the faith. The beast must be fed or he famishes; and he does not hunt husk either, he wants corn. Show him where it is and he'll root for it. Here, then, is the problem in a nutshell: make this Society the crib, fill it with golden ears, and crowd the house. I hope no one will grunt at this simile.

Too much of our time is wasted reading and listening to the reading of papers, and if every one of you were to utter a hearty "amen" at this very minute I should not be surprised. Upon the contrary, I hope you will get such a surfeit to-night that you will never listen to another.

I am also opposed to the wasting of time reading a minute of previous meetings. That, however, we cannot do away with, but for brevity's sake, cut it down three-quarters, anyhow.

In place of the essay I would institute an experience meeting. The President could ask the faithful if they had anything to communicate that was fresh and good. This would open the ball early, thus giving each a chance to contribute whatever they had to the common fund. Every one of us cracks a nut or two during the month that contains a rich, nutritious kernel, and when thirty or forty such are spread before us we have a feast. To this end let the individual members of this Society constitute themselves observers of "clinical confirmations," only recording such as are unquestionable and uncontaminated; that is, confirmations where the single remedy has been used. A year of such research would collect a *wealth* of knowledge for practical work. We need not seek to do original labor. My honest conviction is that we know too little of the material we already have. What is needed is good, palatable preparation, so that it can be assimilated. Most of us are starving with plenty around. We clutch at a new book as though half famished, and read that the one dec. to the third cent. only can be relied upon. Then comes another, and to our utter amazement, we have been hugging the corpse of a fatal error. *Nothing* but the infinitesimals are fit for the sick. Could *anything* sound more like a Chinese orchestra? From this confounding din must our practice be extricated, and, guided by the beacon that any man can see, *Similia similibus curantur*, it *can* be extricated; that is Homœopathy, and this Society should stick to its seal there and stand by it.

I think I hear you say, The ideas *are* familiar, indeed, quite threadbare. We know all that; tell us something new. Do you know it all? Is it familiar? If so, why do we have Arndt's *System*, Lilienthal's *Therapeutics*, and a host of other works? Do not such publications detract from the study of materia medica—do more to prevent the selection of drugs according to the totality of disease symptoms than we are willing to admit? Works on practice, so called, have no business to deal with materia medica; it's a separate and distinct branch of medical art. The time spent in preparing such literature should have been applied to our means of healing the sick. And *had* it been so applied we would to-day have a REPERTORY to our vast armamentarium that would serve other purposes than filling space upon our book shelves.

In the branches of medical literature common to all schools we do not excel the allopath, hence there is no field open to us but materia medica. In this field our methods are our distinguishing features, and we should confine our labors to that alone. Materia medica we have, *repertory* we have not, and right here lies the need of to-day. Solve the problem and confer a blessing where it is most needed.

The hour of our meeting that should be the most profitable is very often crowded out altogether—I mean "Diseases of the Mouth." It should *never* be so. The reports and discussions should be concise, clear cut, and comprehensive; the sounds should never be uncertain. What benefit is it to you to hear that diarrhœa prevails during the month of August, and that Podo. is the remedy? Not a whit. If I come here and tell you, painless, profuse, watery stools, smelling like carrion, loud gurgling in the bowels before the stool, gagging and empty retching, worse morning, night, and hot weather, great thirst and cold limbs, were promptly relieved by Podo.$^{3, 30, 200, \text{ or } 100}$, as the case may be, I have told you something, probably not new, but very important.

Where is the good of writing a paper after the manner in vogue and followed by what is called indications for the remedies? None whatever. Can treatment by a homœopath be given in any case until the totality of the sick person's symptoms has been taken? I think not. Of course, I appreciate the great difficulty of always obtaining a totality of symptoms. A screaming child or a stupid person furnish very poor subjects for the illustration of similia, but screaming children and stupidity are not the majority of our victims; quite the contrary. And since we have been commissioned homœopathic physicians and Homœopathy means *Similia similibus curantur*, the laborious task of gathering the totality of signs and finding the drug similar is self-imposed, and we cannot be true to the trust if we practice anything short of it.

The cases reported from practice should meet the requirements of a strict rendering of the law. But, says my modern homœopath, it does not meet all the emergencies; it's a rule of practice, not the law. Who does not remember the early crudities of electrical appliances? Who dreamed of the potentiality of that subtle force? One wire with ponderous gin-mill appliances transmitted messages short distances. There were incredulous men then, as now, and they stood doubting. Yet to-day perfected instruments and further knowledge of the mysterious power has wrought the duplex, quadruplex, cable, motive force, electric light, and telephone. Were these wonderful achievements accomplished by the men who stood by content with what they had? Not at all. *Doubting* men drag down, *credulous* men build up. So with Homœopathy; it's as much a law of nature as electricity, and as capable of manipulation. The possibilities underlying it are as susceptible of development and appliance as the captive lightning. It will not do, gentlemen, to decry it because it is not yet perfected, but rather, instead, work it with the perseverance of an Edison, and where *he* has *lighted* the world *you* will liven it.

Does Ipecac cause and cure certain well-known symptoms? Certainly; you all believe it. Then it's the *law* of cure and not a rule, for what is true of Ipecac *must* be true of all drugs. But I am told that there are disease symptoms that are as yet uncovered by drug symptoms. If so, is not the duty before us plain enough—a *work* to accomplish? The next thing is the means to accomplish it. Disease symptoms to cover?—the next thing is to find the means to cover them. Our obligation to the sick demands it of us, and our duty will never be discharged until every substance has been tested to that end. Right here comes a rub. Unless our case is perfectly clear, we are prone to cover by complex prescribing or fall back upon some of the expedients of empiricism, a practice that has brought reproach upon us. We do this for shekels, not for Homœopathy, and soothe a guilty conscience by the assurance that it's what allopaths would do, anyhow. It's a great pity we have such easy exit from a trying situation, else we would hunt for the single curative drug. What therapeutic benefit is derived from complex prescribing? Not a bit; besides, it's unhomœopathic. A lady told me recently that her doctor was in the habit of mixing two or three remedies in the same glass. Spirit of the departed! what kind of a "path" was he? It won't do, gentlemen; it's husk without a grain. There need be no quarrel upon questionable points or disputed grounds. When each and all of us become too conscientious to commit a willful error, the potency and alternating questions will settle themselves. I'm neither prophet nor son of a prophet, but I venture to record right here that when such time comes you will prescribe the single remedy and higher potencies. If we are to continue homœopathic physicians we must cease hankering after the flesh pots of false gods, and if virtue hath its own reward, then the man or woman who *knows* the law of similars to be true and adheres to it throughout the domain of medical practice will not go unrewarded. "Homœopathy repays its votaries," says Reichelm, and he spake the truth. To be sure, the acts of gathering the symptoms and selecting the remedy do not give forth the grandiloquent sounds of iridectomy in glaucoma, tracheotomy in croup, tracheloraphy in hypertrophied cervix, etc., but the results are quite as good if not better; *besides, there is a something in the heart of every man who studies his case well that pats him on the back and feathers his cap with success.*

I have said that we need not quarrel over the potency question; but we do. The best preparation of a remedy to be used is still an open one. Notwithstanding the fact that the thirtieth was considered the best, the majority of us stick to the lower and lowest dilutions, utterly ignoring the possible potentiality of high dilutions. Now there is room for solemn protest against the condemnations so often hurled at high potencies by those who never use them. While I use all potencies, I can say that the most gratifying exclamations have come from those who have received the higher potencies. I also observe that the men of greatest faith in the law, the strictest followers of the rule, are those who use the high and highest potencies. You do not hear *them* proclaiming the failure of Homœopathy. *They* do not prate about *vis medicatrix naturæ*, but, with an apparent zeal and enthusiasm born of success, *they defend the faith.*

The moment any man tries to balance the scale of his practice by the ponderous porportion of his dose, *just that moment will he find the health end of the beam jerked higher than a kite.*

Recently a lady was sick with abdominal pains, caused by incarcerated flatus that gathered in the left upper abdomen. The pains were accompanied by continuous uterine hemorrhage, complete anorexia, etc., which had lasted nine weeks. Ars. iod. in one glass and Dioscorea in another were being taken without relief. A review of the case seemed to clearly indicate Lycop., and eight powders of the 200th were prescribed. The first powder relieved her of pain. Sac. lac. followed for twenty-four hours. The hemorrhage continued. China$^{87m}$, eight powders, followed by Sac. lac. In six hours after the first powder the hemorrhage ceased, appetite returned, and in five days the lady journeyed fifty miles by rail. Was the result due to *vis. med. nat.*, or did the woman only want a chance to get well after the discontinuance of complex prescribing in low dilutions? Choose whatever conclusion you will.

Another lady complained of a train of symptoms I will not here enumerate who had been prescribed for a number of times by a Hahnemannian homœopathist without relief. A comparison of her symptoms with those of Lil. tig. caused that remedy to be selected in the 200th potency. The symptoms were relieved by the remedy and no other. Now, what does it prove? *It proves that when we hit the remedy success follows.* It also proves that high dilutions have as good a right to be relied upon as the low.

One of the great troubles of the present is that many of us have become, or think we have become, greater than our master. The cry is that Homœopathy *must* be placed upon a *scientific* basis. Boston is to-day the proud possessor of one such man. According to this wise man of the East, the superstructure of homœopathic proving is incomplete, the *animal* world has not been sufficiently drawn upon, and this great microscope man would have molluscs, radiates, articulates, etc., lay tribute upon the altar of drug pathogenesy. In point of originality of thought and brilliancy of conception, the genius of the now almost forgotten sage of Cothen *pales* when compared with this nineteenth century Moses who would lead the floundering hosts of Homœopathy into the Promised Land of medical science. How edifying and how elevating to be able to confirm the tearfulness of Puls. or the restlessness of Ars. in that luscious lump of protoplasmic pulp—the oyster. Methinks that long before the flood-gates of grief were opened or the o'erwrought nervous system began to writhe, that temptation would get the better of science, and, *presto*, the whole business be swallowed up sans salt, pepper, or vinegar. Suppose, too, that a modern homœopath should attempt to verify his experience with saturated solutions of Chlorate of Potash or ten grain solutions of Arg. nit. upon the pharyngeal erysipelatous œdema of the turtle. Wouldn't the turtle snap at the game if he was that kind of a T.? or the surgical homœopath who would cut out the turbinated nasal bones to increase the breathing capacity of some batrachian asthmatic. What conclusion would that frog jump at? That Homœopathy had indeed been placed upon a scientific basis, and that the bones of Hahnemann were rattling in their tomb.

Z. T. Miller, M. D.

